# Using Documentary Short Film for Health Impact: An Example for Supporting Family Caregivers

**DOI:** 10.1093/geroni/igab046.725

**Published:** 2021-12-17

**Authors:** Janice Bell, Jessica Zitter

**Affiliations:** 1 University of California, Davis, Sacramento, California, United States; 2 Highland Hospital, Oakland, California, United States

## Abstract

Storytelling through film is a powerful tool with potential to improve understanding, spark discussion, shape perceptions of health and illness, and influence related behavior. We developed a film discussion guide for the documentary short film Caregiver: A Love Story. The 24-minute film follows the experience of a man who leaves his job to become the primary caregiver of his 59 year-old wife, who opts out of non-beneficial chemotherapy, choosing instead to remain at home with hospice support. The 2-hour program was facilitated by an experienced social worker and offered on five different days/times using Zoom video (n=60 total attendees; 9-15/ session). At each session, we showed the film, discussed self-care and caregiver resources, and fielded a survey to assess satisfaction, format acceptability and session impact (response rates 67-100%/session). Attendees liked the session format (90%); found the film relevant to their situations (80%); intended to look for new professional resources (79%); were motivated to do something different(71%); learned something new (64%); and intended to ask for more help from family or friends (64%). Many commented that they preferred the video meeting format over in-person meetings. The film viewing and discussion format is acceptable and accessible to family caregivers who may otherwise not be able to attend given competing demands. This format also has potential to improve support access to resources. Extensions to this work are planned to tailor the film discussion guide for health care providers and students working with family caregivers across inpatient, outpatient and hospice settings.

